# Correction: Evolution of an ancient protein function involved in organized multicellularity in animals

**DOI:** 10.7554/eLife.14311

**Published:** 2016-01-21

**Authors:** Douglas P Anderson, Dustin S Whitney, Victor Hanson-Smith, Arielle Woznica, William Campodonico-Burnett, Brian F Volkman, Nicole King, Joseph W Thornton, Kenneth E Prehoda

Anderson DP, Whitney DS, Hanson-Smith V, Woznica A, Campodonico-Burnett W, Volkman BF, King N, Thornton JW, Prehoda KE. 2016. Evolution of an ancient protein function involved in organized multicellularity in animals. *eLife* **5**:e10147. doi: 10.7554/eLife.10147.

Published 07 January 2016

The order of the co-corresponding authors was incorrect. The correct author order in full is: Douglas P Anderson, Dustin S Whitney, Victor Hanson-Smith, Arielle Woznica, William Campodonico-Burnett, Brian F Volkman, Nicole King, Joseph W Thornton, Kenneth E Prehoda

The article has been corrected accordingly.

